# Meteorological and environmental factors associated with the spatiotemporal risk of severe fever with thrombocytopenia syndrome in Japan: A prefecture-level time-series modeling study

**DOI:** 10.1016/j.onehlt.2026.101547

**Published:** 2026-08-14

**Authors:** Fangyu Yan, Sophearen Ith, Noriko Kitamura, Yu Takizawa, Taro Kamigaki, Motoi Suzuki, Ken Maeda, Daisuke Yoneoka

**Affiliations:** a Department of Infectious Disease Surveillance, National Institute of Infectious Diseases, Japan Institute for Health Security, Tokyo, Japan; b Department of Epidemiology, National Institute of Infectious Diseases, Japan Institute for Health Security, Tokyo, Japan; c Center for Infectious Disease Epidemiology, National Institute of Infectious Diseases, Japan Institute for Health Security, Tokyo, Japan; d Department of Veterinary Science, National Institute of Infectious Diseases, Japan Institute for Health Security, Tokyo, Japan; e Department of Global Health Policy, Graduate School of Medicine, The University of Tokyo, Tokyo, Japan

**Keywords:** Severe fever with thrombocytopenia syndrome, Tick-borne disease, Generalized additive model, Meteorological factors, Environmental factors, Non-linear association

## Abstract

**Background:**

Severe fever with thrombocytopenia syndrome (SFTS) is an emerging tick-borne zoonotic disease with a high case fatality rate and expanding geographic distribution in Japan. Comprehensive evaluations that integrate meteorological, environmental, and demographic factors remain limited. This study aimed to quantify these associations.

**Methods:**

We conducted a nationwide prefecture-level time-series analysis using weekly SFTS surveillance data in Japan from March 2013 to December 2025. A generalized additive mixed model adjusting for nonlinear seasonality and long-term trends was used to assess (1) the associations between SFTS risk and meteorological, environmental and demographic factors, and (2) the value of the factor at which risk become highest.

**Results:**

A total of 1237 SFTS cases were reported. Mean temperature and precipitation showed inverted U-shaped associations with SFTS risk, with peak risks observed at 21.8 °C (relative risk [RR]: 1.78, 95% confidence interval [CI]: 1.26–2.52) and 156.6 mm (RR: 1.30, 95% CI: 1.04–1.62), respectively. Atmospheric pressure and forest coverage were positively associated with SFTS risk, with maximum RRs of 2.06 (95% CI: 1.44–2.93) at 1027.1 hPa and 6.58 (95% CI: 4.95–8.74) at 0.84, respectively. Mean altitude showed an inverse association, with the highest RR observed at 45.0 m (RR: 3.99, 95% CI: 1.81–8.76).

**Conclusions:**

The increasing number and geographic expansion of SFTS cases highlight the growing public health importance of this disease in Japan. Our findings improve understanding of factors associated with SFTS transmission and may inform future risk assessment and public health preparedness.

## 1 Introduction

Severe fever with thrombocytopenia syndrome (SFTS) is an emerging tick-borne zoonotic disease caused by the SFTS virus (SFTSV), a negative-sense RNA virus belonging to the genus *Bandavirus* within the family *Phenuiviridae* [1]. The virus is primarily transmitted to humans through the bite of infected ticks, particularly *Haemaphysalis longicornis*, although human-to-human and animal-to-human transmission through contact with infected blood or body fluids has also been documented [2]. The case fatality rate is high (approximately 20% overall and considerably higher among older adults), and no licensed vaccine is currently available [3]. Due to number and geographic expansion, relatively high case fatality rate and the lack of effective vaccines, SFTS is recognized as an important emerging public health threat worldwide.

SFTS was first identified in China in 2009 and has since been reported in other Asian countries, including Korea, Japan, Thailand, Myanmar, and Vietnam [4], [5], [6], [7], [8]. In Japan, the first SFTS case was identified in Yamaguchi prefecture in 2013, with the annual notification rate increased from 3.12 in 2013 to 10.46 in 2023 per ten million population [3], [9]. Until recently, reported cases had been largely confined to the warmer south-western regions of the country, including Kyushu, Shikoku, and Chugoku regions. In 2025, SFTS case was reported in Hokkaido (the northernmost region of Japan), suggesting a substantial northward expansion of the disease distribution [10], [11]. One possible explanation is the long-distance dispersal of ticks by migratory birds [12], which can carry SFTSV-infected *H. longicornis* along their flyways into previously unaffected regions. This poleward shift parallels the climate-driven distribution expansion of *H. longicornis* and related ixodid vectors reported across the temperate Northern Hemisphere [13]. It is progressively exposing populations in previously low-risk regions, including areas with limited clinical familiarity with SFTS, insufficient diagnostic capacity, and limited public awareness regarding tick-bite prevention.

SFTS is considered a climate-sensitive infectious disease, as appropriate meteorological and environmental conditions can accelerate tick's development, prolong survival, and promote questing activity, thereby promoting the replication and transmission of pathogens [14]. A meta-analysis reported that temperature, humidity, precipitation, sunshine duration, and atmospheric pressure were significantly associated with SFTS incidence, although the magnitude and direction of these effects varied across regions [15]. However, most of the evidence originates from mainland China. Other factors such as altitude, land cover type and vegetation were reported as risk factors because they affect tick habitat suitability, microclimatic conditions, and the distribution of host animals [16], [17]. Social-economic factors such as gross domestic product (GDP) and population density are also reported as important determinants of SFTS risk due to greater outdoor exposure of larger populations to ticks [16], [18], [19]. Japan offers a particularly informative setting for examining how these determinants combine. Approximately two-thirds of Japan's land area is forested [20], representing one of the highest forest coverage proportions among high-income countries, and its population is the most aged in the world, a demographic group at markedly elevated risk of severe SFTS outcomes [21].

Quantitative evidence on factors associated with SFTS risk in Japan was limited. Domestic studies have been geographically restricted or descriptive in analysis scope, have examined candidate factors separately, and have not adequately captured potential non-linear exposure–response relationships [3], [22], [23]. As the geographic distribution of tick-borne disease continues to shift under ongoing climatic and ecological change, public health adaptation depends on quantifying the meteorological, environmental, and demographic conditions under which SFTS occurs, rather than only describing where it has previously been observed [24]. We therefore conducted a nationwide, prefecture-level time-series analysis covering 2013–2025 to quantify how these factors jointly shape SFTS risk in Japan, identify the exposure ranges at which risk is greatest, and examine whether these relationships have changed over the course of the epidemic. The resulting evidence is intended to inform climate-adaptive surveillance and prevention in Japan and in comparable ageing societies across the Western Pacific.

## 2 Methods

### 2.1 SFTS and population data

SFTS is a category IV infectious disease under the Infectious Diseases Control Law in Japan, and all the laboratory confirmed case are mandated to report to National Epidemiologic Surveillance of Infectious Diseases (NESID) [3]. Weekly counts of reported SFTS cases aggregated at the prefectural level from epidemiological week (EW) 13, 2013, when the first human SFTS case was reported in Japan, to EW 52, 2025 were obtained from Infectious Disease Weekly Report (IDWR) of National Institute of Infectious Diseases, Japan Institute for Health Security [25]. Yearly population data at the prefectural level from 2013 to 2025 were obtained from the Statistics Bureau of Japan based on the national census and intercensal estimates [26]. Annual population estimates were assumed to be constant within each year.

### 2.2 Meteorological, land use and demographic data

Variables included in our analysis were selected based on previous studies and findings from studies conducted in other countries [15], [27], [28], [29], [30], [31], [32], [33], [34], [35]. Weekly meteorological variables including temperature (°C), relative humidity (%), precipitation (mm) were obtained from the European Center for Medium-Range Weather Forecasts (ECMWF) fifth-generation global atmospheric reanalysis (ERA5-Land) [36]. ERA5-Land is a global land-surface reanalysis dataset that provides climate data at fine spatiotemporal resolution on a 0.1° × 0.1° grid. Hourly data were aggregated into daily minimum, mean and maximum temperature (°C), mean relative humidity (%) and total precipitation (mm). Daily mean atmospheric pressure (hPa), mean wind speed (m/s) and total sunshine duration (hours) were obtained from representative monitoring stations for each prefecture at the Japan Meteorological Agency (JMA) [37]. The daily data were then further summarized into weekly prefecture level. Additional factors included forest coverage, dry agricultural land cover, population aged ≥65 years, and altitude. Forest coverage area data were obtained for the years 2012, 2017, and 2022 [38]. To generate annual estimates from 2013 to 2025, linear interpolation was applied within each prefecture. Dry agricultural land coverage area data were obtained annually at the prefectural level from official national statistics [39]. Both paddy fields and dry agricultural land were available, and we used only dry agricultural land as ticks are less likely to inhabit water-saturated environments. Forest coverage (proportion) and agricultural land coverage (proportion) was defined as the proportion of forest area and dry agricultural land relative to the total area of each prefecture. Prefecture-level area data were obtained from national statistics. Age group specific population data by prefecture was obtained from Statistics Bureau of Japan [39]. We used the proportion of population aged ≥65 years, and the prefecture-level mean altitude (m) from Geospatial Information Authority of Japan [40]. Detail of data sources, variable definitions, and their temporal and spatial resolutions are summarized in Supplementary Table S2. Pairwise Pearson correlations among all variables were assessed to evaluate potential collinearity, and no severe collinearity was observed with correlation coefficients below 0.6 (Fig. S1).

### 2.3 Statistical analysis

Descriptive analyses were performed for all variables, including calculation of the mean, median, standard deviation (SD), minimum, and maximum values. Then we used a generalized additive mixed model (GAMM), assuming a negative binomial distribution to account for overdispersion in SFTS cases. The baseline model includes seasonal terms, long-term trend terms, and a prefecture-level (Gaussian) random effect. Climatic variables were then added to the model, followed by a full model including both climatic and non-climatic variables (Table S4). The best model was selected based on the Akaike Information Criterion (AIC), while also maintaining biological plausibility and interpretability. The final model was given by:

$$Y_{i,t}∼\mathrm{NegBin}\left(\mu_{i,t},\phi_{i}\right)$$

$$\log\left(\mu_{i,t}\right)=\alpha +\log\left(P_{i,t}\right)+s\left({\mathrm{week}}_{t},\mathrm{bs}=\mathrm{cc},k=6\right)+s\left({\mathrm{year}}_{t},k=6\right)+b_{i}+\sum_{m=1}^{6}s\left(C_{m,i,t},k_{m}\right)+\sum_{n=1}^{N}s\left(Z_{n,i,t},k_{n}\right),$$

where $Y_{i,t}$ denotes the number of SFTS cases in the prefecture i during week t, assuming a negative binomial distribution with the mean $\mu_{i,t}$ and dispersion parameter $\phi_{i}$; $P_{i,t}$ is the number of population in the prefecture i included as an offset; $s\left({\mathrm{week}}_{t},\mathrm{bs}=\mathrm{cc},k=6\right)$ is a smooth function of week of the year, modeled using a cyclic cubic spline (denoted as cc in s() function) with a basis dimension of 6; $s\left({\mathrm{year}}_{t},k=6\right)$ denotes a thin plate splines function of calendar year with a basis dimension of 6; $b_{i}\;$is the prefecture-specific random effect to account for heterogeneity across prefectures;$\;\sum_{m=1}^{6}s\left(C_{m,i,t},k_{m}\right)$ is the smooth functions of meteorological factors, including mean temperature (k = 4), relative humidity (k = 3), total precipitation (k = 3), total sunshine duration (k = 4), atmospheric pressure (k = 4), and mean wind speed (k = 7). $\sum_{n=1}^{N}s\left(Z_{n,i,t},k_{n}\right)$ is the smooth functions of other factors, including forest coverage (k = 5), mean altitude (k = 6), dry agricultural land coverage (k = 4), and the proportion of the population aged 65 years or older (k = 3). Using the overall mean of each variable as the reference, relative risks (RRs) and 95% confidence intervals (CIs) were estimated. Lastly, for each variable, we reported the maximum RR and its corresponding exposure value, along with the estimated RRs at the 5th and 95th percentiles, representing the lower and upper extremes of the exposure distribution. All of the statistical analysis implemented in R version 4.5.3.

### 2.4 Sensitivity analyses

Several sensitivity analyses were conducted to check the robustness of the results. First, minimum and maximum temperature were used as a covariate instead of the mean temperature. Second, we stratified the study period into two phases in order to examine the time-dependent covariate effects: the early period from March 2013 to December 2018, and the late period from January 2019 to December 2025. Third, a sensitivity analysis restricted to western Japan, where SFTS cases were concentrated, was conducted. Forth, to assess the relative contribution of each variable, we conducted a leave-one-variable-out analysis and evaluated changes in the AIC of model.

### 2.5 Role of the funding source

The funders had no role in study design, data collection, data analysis, data interpretation, or writing of the report.

## 3 Results

**Fig. 1:**
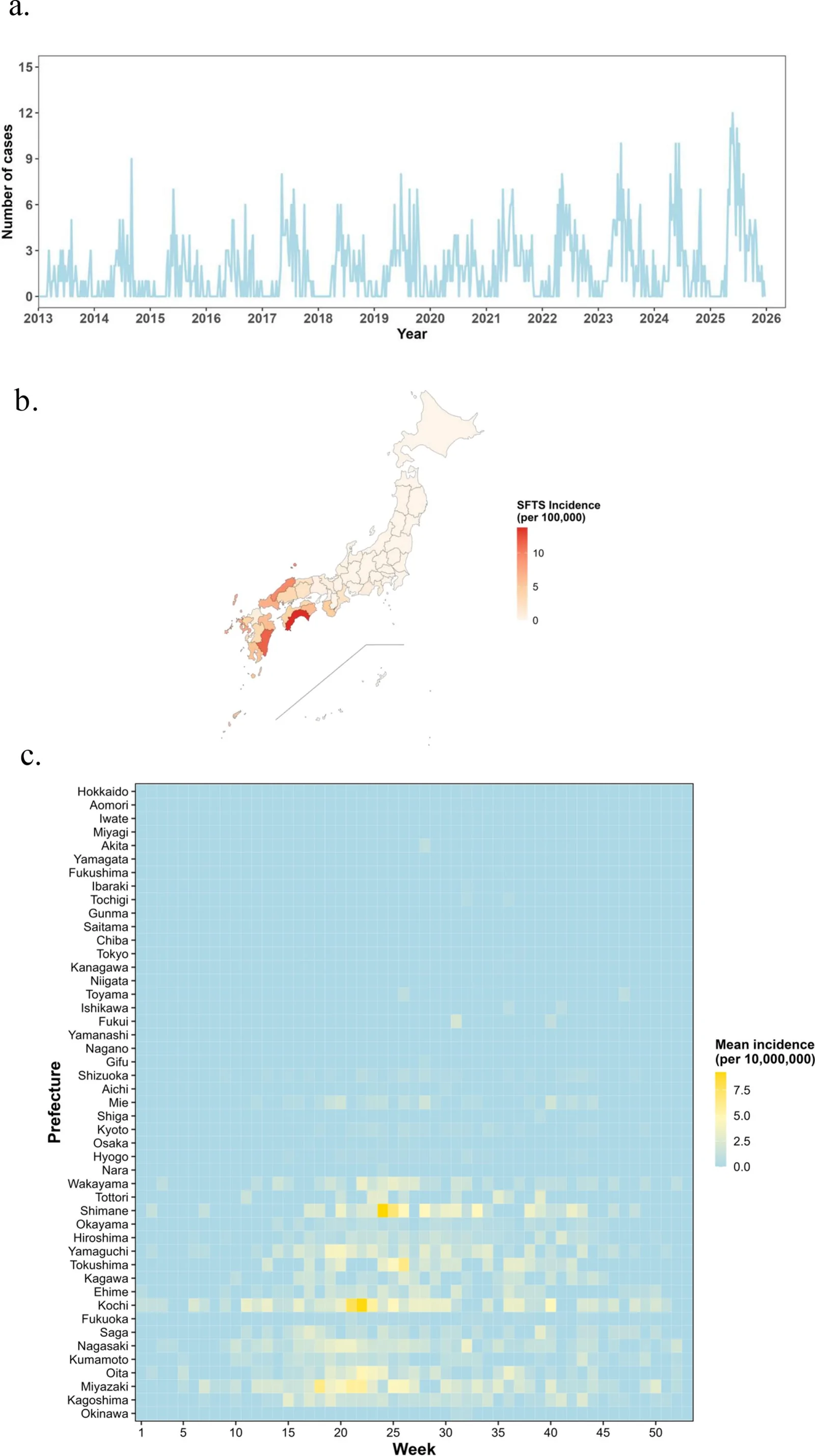
Temporal and spatial distribution of severe fever with thrombocytopenia syndrome (SFTS) cases in Japan, 2013–2025. (a) Epidemic curve of weekly SFTS case; (b) Geographic distribution of overall SFTS incidence per 100,000 population; (c) Weekly mean SFTS incidence per 10,000,000 population by prefecture.

A total of 1237 SFTS cases were reported from March 2013 to December 2025. The yearly total number of reported SFTS grows gradually from 48 in 2013 to 178 in 2025. The epidemic curve showed a bimodal seasonal pattern, with a major peak in May and June and a smaller peak in October (Fig. 1a, c and Fig. S5). In 2025, the geographic distribution of SFTS cases expanded northward, with newly identified cases reported in Hokkaido, Akita, Ibaraki, Tochigi and Gifu prefecture (Supplementary Fig. S2). The highest number (124 cases) of accumulated cases were reported from Miyazaki prefecture while the highest incidence (13.8 per 100,000) was in Kochi prefecture (Fig. 1b and Fig. S3). Seasonality patterns were also found in climatic factors (Fig. S4, S5). During the study period, nationwide mean (SD) of weekly temperature, relative humidity, and atmosphere pressure are 13.5 °C (8.6), 77.1% (7.1), and 1008.4 hPa (10.3); respectively (Table 1).

**Table 1:** Summary statistics of severe fever with thrombocytopenia syndrome cases, weekly meteorological and non-meteorological variables in Japan from 2013 to 2025.

Variables	Mean	Median	SD	Minimum	Maximum
Cases	0	0	0.2	0	4
Temperature (°C)	13.5	14	8.6	−11.7	29.7
Precipitation (mm)	37.2	26.7	38.7	0	479.4
Atmospheric pressure (hPa)	1008.4	1010.1	10.3	953.6	1027.1
Relative humidity (%)	77.1	77.3	7.1	42.4	96.9
Sunshine (hours)	38.8	38.4	16	0	91.7
Wind speed (m/s)	2.9	2.8	0.9	0.9	14
Forest coverage(proportion)	0.6	0.7	0.2	0.3	0.8
Mean altitude (m)	361.3	314	214.6	45	1132
Dry agricultural land coverage (proportion)	0	0	0	0	0.2
Population aged ≥65 years (proportion)	0.2	0.2	0	0.1	0.4

SD: standard deviation.

For the spatiotemporal pattern, incidence was concentrated in Chugoku, Shikoku and Kyushu region during week 18 to 26 (Table S1, Fig. 1c). The peak was observed in Shimane Prefecture in week 24, followed by Kochi Prefecture in week 22, with incidence of 9.23 and 9.15 per 10,000,000 population, respectively. Miyazaki and Tokushima Prefectures also showed relatively high incidence in weeks 21 and 26, respectively.

### 3.1 Meteorological factors and SFTS

**Fig. 2:**
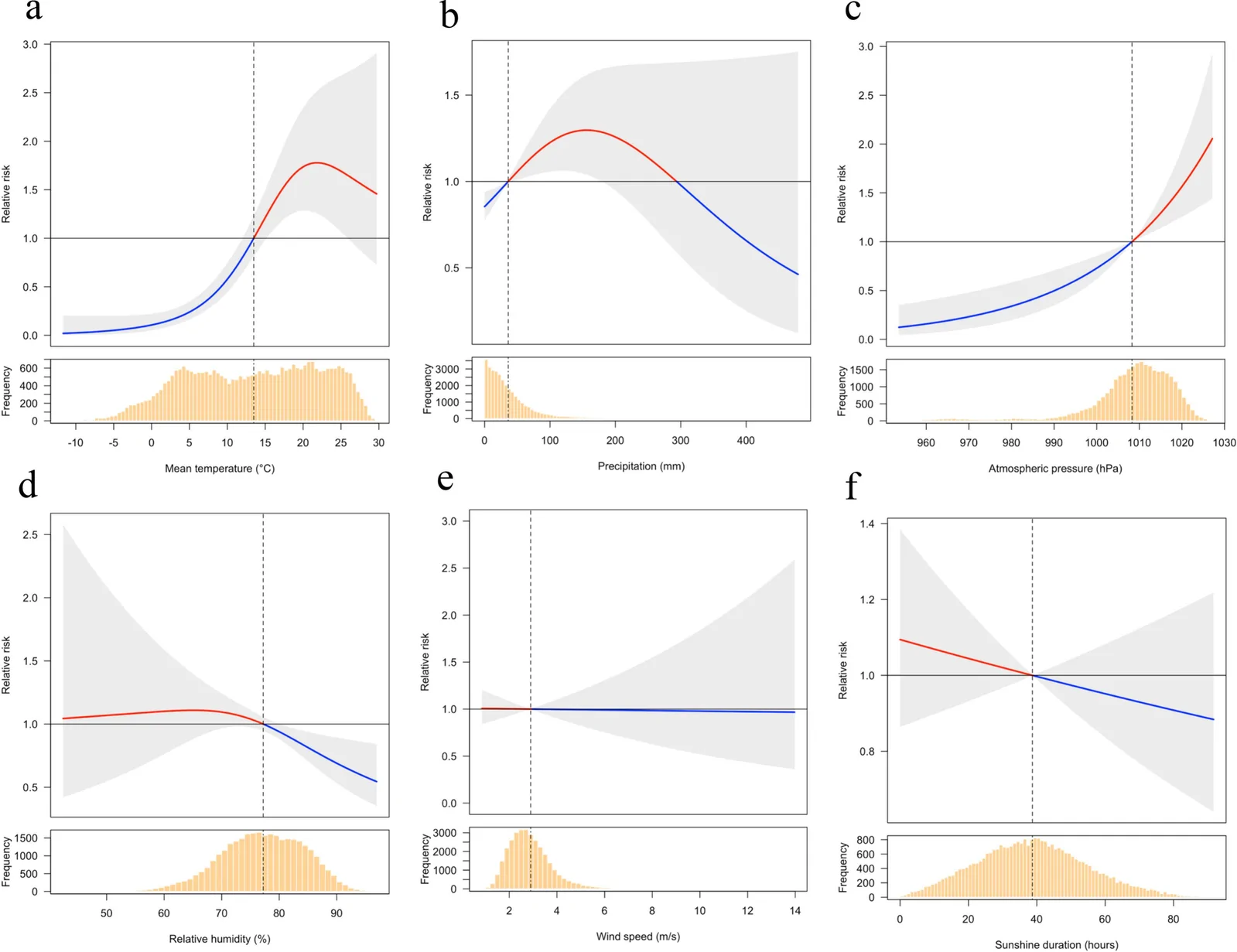
Non-linear relationship between weekly meteorological factors and severe fever with thrombocytopenia syndrome risk. (a) mean temperature; (b) total precipitation; (c) mean atmospheric pressure; (d) mean relative humidity; (e) mean wind speed; and (f) total sunshine duration. The reference value (vertical dashed line) was set at the overall mean of 13.5 °C for temperature, 37.2 mm for total precipitation, 1008 hPa for atmospheric pressure, 77.1% for relative humidity, 2.9 m/s for wind speed, and 38.8 h for sunshine duration. Blue and red solid lines represent decreased and increased relative risks, respectively. Shaded areas indicate 95% confidence intervals. Histograms show the distribution of each variable. (For interpretation of the references to colour in this figure legend, the reader is referred to the web version of this article.)

Compared to the overall mean, weekly mean temperature and total precipitation showed inversed U-shaped association with SFTS risk, with the peak at 21.8 °C (RR = 1.78, 95% CI: 1.26–2.52) and 156.6 mm (RR = 1.30, 95% CI: 1.04–1.62), respectively (Table 2, Fig. 2). Extreme low temperature at the 5th percentile (−0.6 °C) was associated with a lower risk of SFTS (RR: 0.10, 95% CI: 0.04–0.22), whereas extreme high temperature at the 95th percentile (26.2 °C) was associated with an increased SFTS risk (RR: 1.63, 95% CI: 0.98–2.73). Comparing to the mean at 1008.4 hPa, atmospheric pressure was positively associated with SFTS risk, with the maximum RR of 2.06 (95% CI: 1.44–2.93) at 1027.1 hPa. The SFTS risk associated with extreme low (990.5 hPa) and high (1020.5 hPa) atmospheric pressure was 0.51 (95% CI: 0.36–0.71) and 1.59 (95% CI: 1.27–2.00), respectively. The relative risk of humidity peaked at 65.2% (RR = 1.11, 95% CI: 0.88–1.39) compared to the mean of 77.1%. Sunshine duration showed an approximately negative linear association with SFTS risk, with the highest risk observed at 0 h (RR = 1.09, 95% CI: 0.86–1.38), compared with the mean of 38.8 h. Wind speed showed a negative linear association, with risk increasing from the mean value of 2.9 m/s to a maximum at 0.9 m/s (RR = 1.01, 95% CI: 0.84–1.20). However, the observed effects of sunshine duration and wind speed were modest.

**Table 2:** Maximum relative risks and relative risks at the 5th and 95th percentiles, with their corresponding exposure values, for the association between each factor and severe fever with thrombocytopenia syndrome risk.

Variable	Reference value	Maximum RR		Extreme low at the 5th percentile		Extreme high at the 95th percentile	
		Value	RR (95% CI)	Value	RR (95% CI)	Value	RR (95% CI)
Mean temperature (°C)	13.5	21.8	1.78 (1.26–2.52)	−0.6	0.10 (0.04–0.22)	26.2	1.63 (0.98–2.73)
Relative humidity (%)	77.1	65.2	1.11 (0.88–1.39)	64.9	1.11 (0.88–1.40)	88.2	0.73 (0.59–0.91)
Precipitation (mm)	37.2	156.6	1.30 (1.04–1.62)	2.4	0.86 (0.79–0.94)	108.4	1.25 (1.06–1.46)
Sunshine duration (hours)	38.8	0.0	1.09 (0.86–1.38)	12.9	1.06 (0.91–1.24)	66.4	0.94 (0.79–1.11)
Atmospheric pressure (hPa)	1008.4	1027.1	2.06 (1.44–2.93)	990.5	0.51 (0.36–0.71)	1020.5	1.59 (1.27–2.00)
Wind speed (m/s)	2.9	0.9	1.01 (0.84–1.20)	1.6	1.00 (0.90–1.12)	4.5	0.99 (0.86–1.15)
Forest coverage (proportion)	0.63	0.84	6.58 (4.95–8.74)	0.31	0.05 (0.03–0.09)	0.79	3.74 (3.12–4.47)
Mean altitude (m)	361.3	45.0	3.99 (1.81–8.76)	99.6	3.09 (1.70–5.62)	766.0	0.01 (0.00–0.07)
Dry agricultural land coverage (proportion)	0.04	0.04	1.00 (0.89–1.12)	0.01	0.72 (0.63–0.83)	0.12	0.18 (0.12–0.27)
Population aged ≥65 years (proportion)	0.21	0.24	1.06 (0.93–1.21)	0.17	0.73 (0.60–0.89)	0.30	0.86 (0.73–1.00)

RR: relative risk; CI: confidence interval.

### 3.2 Land use, altitude and demographic factors and SFTS

**Fig. 3:**
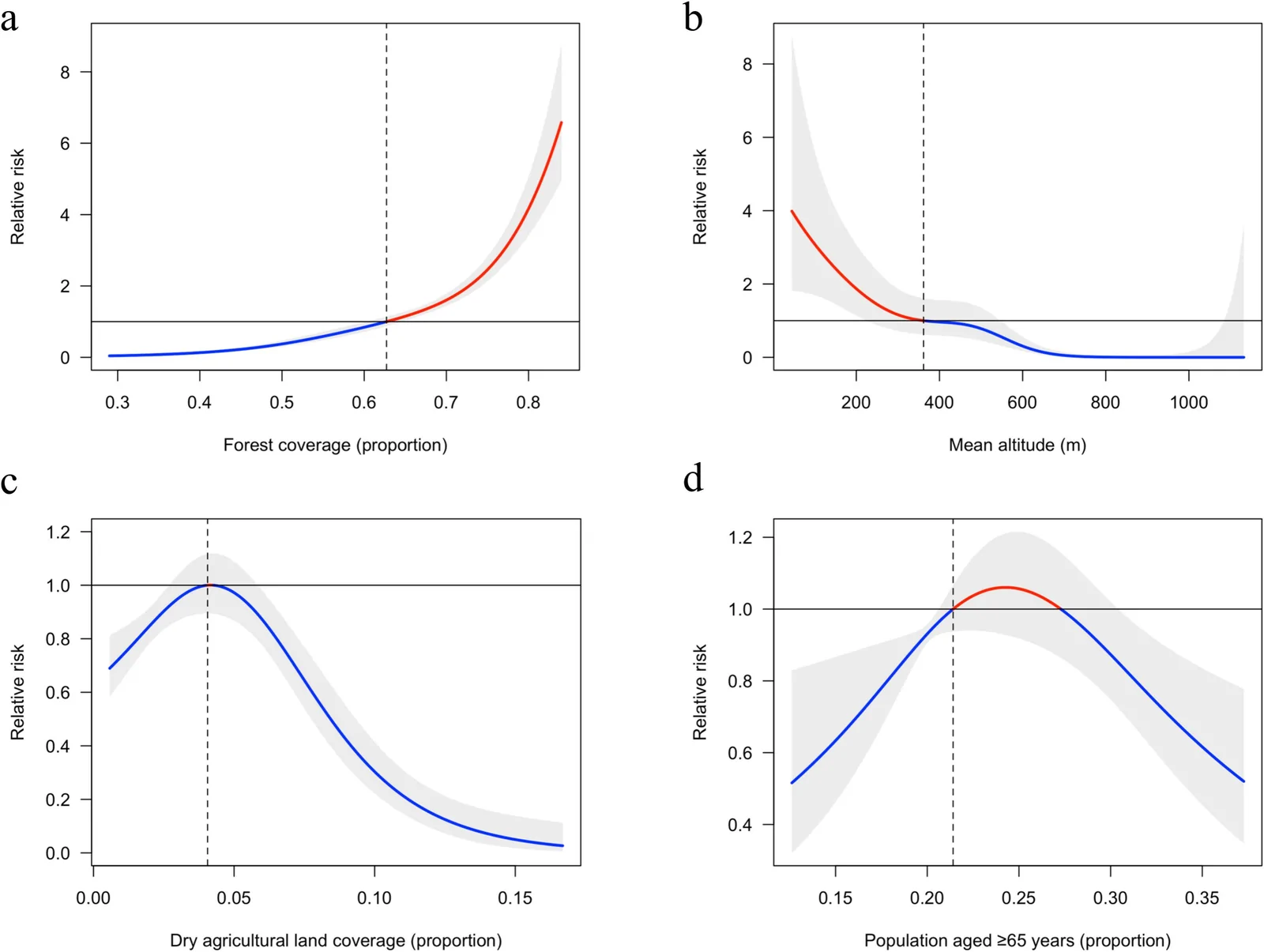
Non-linear relationship between non-meteorological factors and severe fever with thrombocytopenia syndrome risk. (a) forest coverage; (b) mean altitude; (c) dry agricultural land coverage; (d) proportion of population aged ≥65 years. The reference value (vertical dashed line) was set at the overall mean of 0.63 for forest coverage, 361 m for mean altitude, 0.041 for dry agricultural land coverage, and 0.215 for the proportion of the population aged ≥65 years. Blue and red solid lines represent decreased and increased relative risks, respectively. Shaded areas indicate 95% confidence intervals. (For interpretation of the references to colour in this figure legend, the reader is referred to the web version of this article.)

Fig. 3 shows a positive association between forest coverage and SFTS risk with the RR increased sharply beyond the mean value of 0.63 and reached a maximum at 0.84 (RR = 6.58, 95% CI: 4.95–8.74) (Table 2). Compared to the mean (0.63), extreme low forest coverage (0.31) was associated with a lower SFTS risk of 0.05 (95% CI: 0.03–0.09), while extreme high forest coverage (0.79) was associated with a higher risk of 3.74 (95% CI: 3.12–4.47). In contrast, mean altitude showed an inverse association with SFTS risk, with the highest risk was at lower elevations of 45.0 m (RR = 3.99, 95% CI: 1.81–8.76) compared with the mean of 361.3 m. Dry agricultural land coverage showed a non-linear association with SFTS risk, with the risk peaking around the mean value of 0.04 (RR = 1.00, 95% CI: 0.89–1.12) and declining at higher levels. The proportion of the population aged ≥65 years showed an inverted U-shaped pattern, with the RR peaked at 0.24 (RR = 1.06, 95% CI: 0.93–1.21) compared to the national mean 0.21.

### 3.3 Sensitivity analysis

**Fig. 4:**
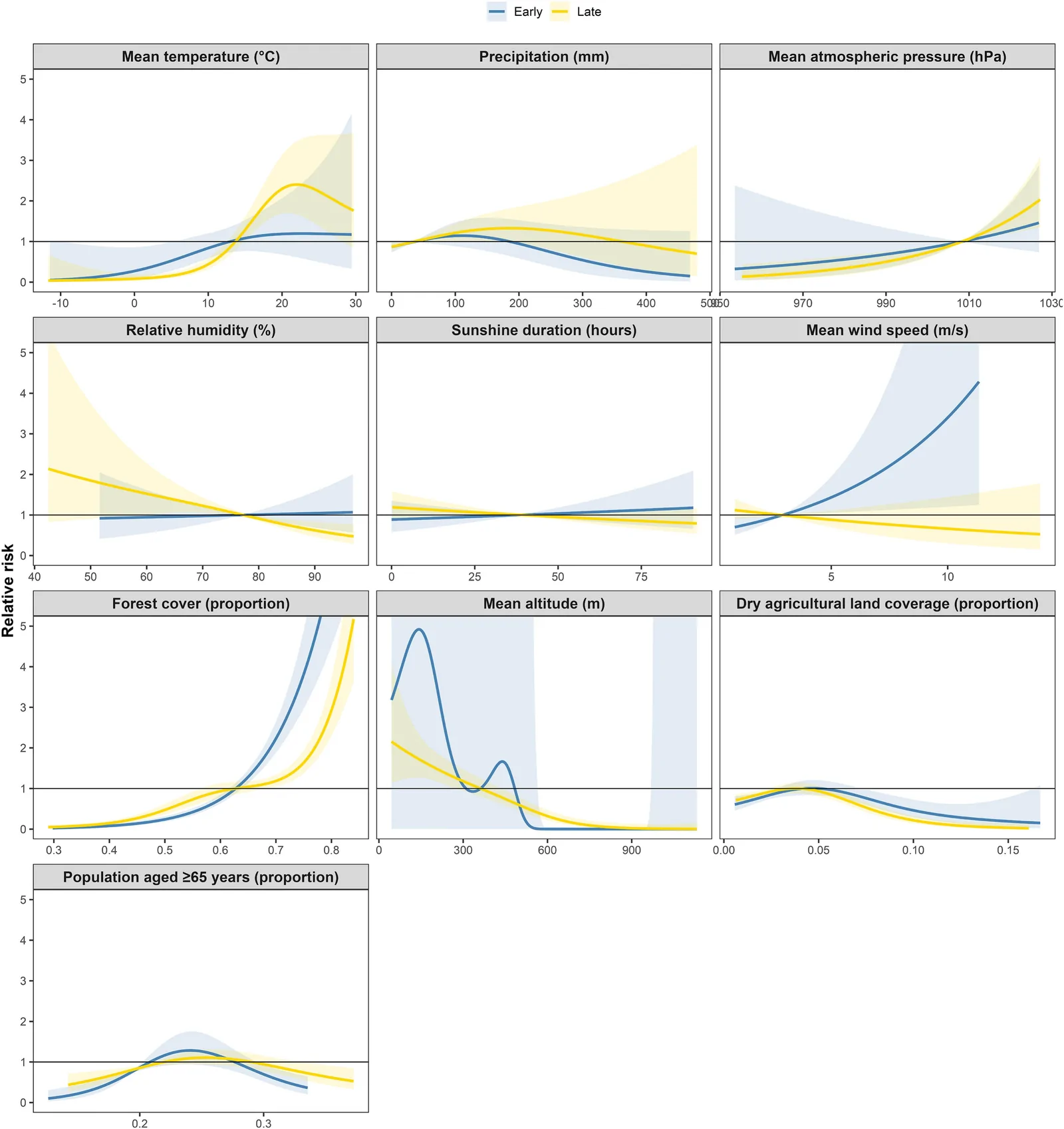
Non-linear relationship between severe fever with thrombocytopenia syndrome risk and meteorological, non-meteorological factors, stratified by early and late period. Note: early defined as March 2013–December 2018 (number of cases: 396), late defined as January 2019–December 2025 (number of cases: 841).

When replacing mean temperature with minimum and maximum temperature, the inverted U-shaped association with SFTS risk remained the same, with the maximum RR was observed at 19.5 °C for minimum temperature (RR = 1.99, 95% CI: 1.38–2.85) and 23.9 °C for maximum temperature (RR = 1.35, 95% CI: 0.99–1.83) (Table S3 and Fig. S7). Time-stratified analyses showed the same inverted U-shaped relationship between mean temperature and SFTS risk for both early and late period. However, the highest relative risk associated with mean temperature was observed at 22.7 °C in the early period (RR = 1.20, 95% CI: 0.59–2.43), compared to 22.0 °C in the late period (RR = 2.40, 95% CI: 1.67–3.47) (Fig. 4). For other factors, the overall shapes of the associations were largely consistent between the early and late periods, but the trend was differed among relative humidity, sunshine duration and wind speed. Geographical stratification analysis restricted to western Japan showed consistent associations between SFTS risk and most factors, although the association with mean wind speed changed from negative to positive (Fig. S8). The estimated effect of mean altitude became stronger, with the highest risk observed at 82.0 m (RR = 4.62, 95% CI: 2.77–7.69), whereas the risks associated with other covariates were attenuated compared with the main analysis (Table S4). Forest coverage was identified as the most important factor in the model, as indicated by the largest increase in AIC (ΔAIC = 181.6) after its removal from the final model (Fig. S8). This was followed by mean altitude (ΔAIC = 90.2) and dry agricultural land coverage (ΔAIC = 52.8).

## 4 Discussion

Our study described the recent epidemiology of SFTS in Japan and investigated the associations between meteorological and environmental factors and SFTS risk in Japan using a 13-year prefecture-level time-series analysis. The use of non-linear functions enabled us to characterize complex exposure–response relationships and to identify potential thresholds at which meteorological and environmental factors may most strongly influence SFTS risk. We observed a recent increase in SFTS cases and a continued northward geographic expansion in Japan, together with distinct non-linear associations of mean temperature, precipitation, atmospheric pressure, forest coverage, and mean altitude with SFTS risk. Temperature and precipitation showed inverted U-shaped associations with SFTS risk, with peak risks at 21.8 °C and 156.6 mm, respectively. The recent increase and geographic expansion of SFTS highlight the need for strengthened public health awareness and preparedness, particularly as outdoor recreational activities and human interactions with tick habitats continue to increase worldwide.

We observed an inverted U-shaped association between temperature and SFTS risk, with the peak 21.8 °C. Temperature has been one of the most extensively investigated climatic factors in previous studies [15]. Previous studies in China have reported similar inverted U-shaped associations between mean temperature and SFTS risk, with peak temperatures range from 17.5 °C to 23 °C [[27], [28], [30]]. Differences in peak temperature across studies may reflect variations in geographic setting, statistical modeling approaches, and the combination of factors included in the analysis. In addition, the time-stratified analyses further indicated that the peak temperature remained largely consistent across periods, whereas the magnitude of the associated RR increased in the later period. One possible explanation is that the substantial increase in SFTS cases during the later period improved the stability and detectability of the exposure–response relationship. In addition, the continued geographic expansion and increasing endemicity of SFTS in Japan may have strengthened the observable association between temperature and transmission risk. These findings suggest potential temporal changes in the impact of climatic factors on SFTS transmission dynamics.

The effect of temperature on SFTS risk could be explained by both biological and sociological mechanisms. From a biological perspective, temperature influences ticks' life cycle, including development, survival, reproduction, and host-seeking behavior [41]. Higher temperatures within a tolerable range can accelerate tick development and increase reproductive capacity, leading to a rapid expansion of tick populations thus increases the likelihood of contact between ticks, humans, and wildlife hosts, resulting in facilitating virus transmission [42]. However, our analysis did not incorporate tick abundance or SFTSV prevalence among ticks, which would have provided a more direct vector-level link to human infection risk. Tick surveillance in Japan remains limited, with reported SFTSV positivity in ticks ranges from undetectable levels in Nagasaki [43] to 5.8–7.1% in Kagoshima [44]. Temperature may influence pathogen replication, with higher temperatures potentially accelerating viral multiplication and thereby increasing the likelihood of transmission to animals and humans [14]. Temperature may also contribute to the expansion of suitable habitats for ticks, potentially extending their geographical distribution [42]. From a socioeconomic perspective, temperature influences human and pet animals' outdoor activities, with moderate temperatures promoting agricultural work and leisure activities, increasing the possibility of human or pet animals' exposure to tick bites.

Similarly, we found precipitation also has inverted U-shaped relationship with SFTS risk. Previous studies have reported inconsistent associations between precipitation and SFTS risk. While some studies have observed a positive association between annual precipitation and SFTS risk, with a notable lag of 2–3 months, others have reported a negative association or non-significant relationship between precipitation and SFTS risk [27], [28], [29]. These inconsistent findings may reflect regional differences in climatic conditions, as areas with relatively high humidity may be less sensitive to variations in precipitation. Precipitation may influence SFTS risk through its effects on vector ecology, host dynamics, and human exposure [15]. Moderate rainfall can create favorable microhabitats that enhance tick survival and activity, while also supporting vegetation growth that sustains host populations such as small mammals. In contrast, excessive rainfall may disrupt tick habitats or reduce host activity. Additionally, precipitation can alter human behavior like agricultural or outdoor activities, thereby modifying the likelihood of human–tick contact and subsequent transmission risk [19].

Our study also found a positive association between atmospheric pressure and SFTS risk in the main analysis. However, the association was attenuated and no longer statistically significant in the sensitivity analysis restricted to western Japan, suggesting that atmospheric pressure had a relatively modest contribution on SFTF risk. Previous studies have identified atmospheric pressure as a key climatic predictor of SFTS incidence, although several reported an inverse association with SFTS risk [31], [32]. Higher atmospheric pressure could be associated with stable weather conditions that promote outdoor activities, so that increasing human exposure to tick habitats. Previous studies have also reported associations of SFTS risk with relative humidity, wind speed, and sunshine duration, although these factors were not statistically significant in our analysis [18], [28], [31], [33], [34].

Consistent with previous studies, we observed a positive association between forest coverage and SFTS risk.Forest may provide suitable habitats for ticks and wild animal hosts that maintain the natural transmission cycle of SFTSV, thereby facilitating viral circulation in these areas. Increased human interaction with the forest may further increase opportunities for exposure to infected ticks [17], [23], [35].*H. longicornis* distribution study in Japan found its population positively associated with forest connectivity, deciduous broad-leaf forest, and raccoon distribution but negatively associated with rice paddy field area, consistent with our finding of declining SFTS risk at higher dry agricultural land coverage [45]. We also found a significant inverse association between altitude and SFTS incidence, in line with earlier reports [23], [29]. Altitude may shape SFTS risk through its effects on meteorological conditions, land use, vector and host ecology, and human occupational and recreational activities.

This study has several limitations. First, the prefecture of cases was defined based on the reporting prefecture rather than the suspected prefecture of infection. This may introduce exposure misclassification. However, the difference between reported and suspected infection prefectures was minimal, with only around 4.3% (51/1185) of cases showing inconsistency, suggesting that the impact on our findings is likely limited [24]. Second, we did not account for potential lag effects between meteorological factors and SFTS incidence. Delayed effects are plausible given the biological processes of tick development, virus transmission and incubation period. In addition, model selection relied on the AIC, which reflects relative goodness of fit rather than predictive accuracy. Future studies should focus on developing predictive models and evaluating their performance through appropriate internal and external validation. Finally, environmental variables were measured at the prefecture level, with three meteorological data derived from a single monitoring station per prefecture. Future studies incorporating lagged meteorological effects and finer-resolution environmental data may further improve the accuracy of risk estimation.

In conclusion, our findings demonstrate that meteorological and environmental conditions play an important role in shaping SFTS risk in Japan. Our study underscores the multifactorial nature of SFTS transmission, likely reflecting the combined influence of vector ecology, host dynamics, and human exposure patterns. Our results provide epidemiological evidence that may support risk stratification, strengthen surveillance, and inform more targeted prevention strategies in Japan and other SFTS-endemic settings in the Western Pacific region.

## Data sharing statement

All data used in this study were obtained from publicly accessible databases. Detailed information on data sources, URLs, and data processing procedures is provided in the Supplementary Table S2. The analytical code of the current study is available from the corresponding author (DY) upon reasonable request.

## CRediT authorship contribution statement

**Fangyu Yan:** Writing – review & editing, Writing – original draft, Visualization, Validation, Methodology, Formal analysis, Data curation, Conceptualization. **Sophearen Ith:** Writing – review & editing, Writing – original draft, Methodology, Data curation, Conceptualization. **Noriko Kitamura:** Writing – review & editing. **Yu Takizawa:** Writing – review & editing, Data curation. **Taro Kamigaki:** Writing – review & editing, Supervision. **Motoi Suzuki:** Writing – review & editing, Supervision. **Ken Maeda:** Writing – review & editing. **Daisuke Yoneoka:** Writing – review & editing, Writing – original draft, Supervision, Methodology, Funding acquisition, Conceptualization.

## Declaration of generative AI and AI-assisted technologies in the manuscript preparation process

During the preparation of this work, the authors used ChatGPT by OpenAI to assist with R coding and language polishing. After using this tool, the authors reviewed and edited the content as needed and take full responsibility for the content of the published article.

## Ethics declaration

The author(s) declare(s) that the study does not involve humans nor animal subjects.

## Declaration of competing interest

The authors declare that they have no known competing financial interests or personal relationships that could have appeared to influence the work reported in this paper.

## Data Availability

Data will be made available on request.
