# Supplementary material for: Meteorological and environmental factors associated with the spatiotemporal risk of severe fever with thrombocytopenia syndrome in Japan: A prefecture-level time-series modeling study
Source: One Health. 2026 Aug 14;23:101547. doi: 10.1016/j.onehlt.2026.101547 (PMC13522366; doi:10.1016/j.onehlt.2026.101547)
Supplement: Supplementary file 1 [file mmc1.docx]

**Supplementary documents**

[Table S1. Definition of region among Japanese prefectures	3](#_Toc234962036)

[Table S2. Variables, data sources, and their temporal resolution used in the study.	4](#_Toc234962037)

[Figure S1. Correlation matrix of the meteorological and non-meteorological factors included in the analysis.	6](#_Toc234962038)

[Figure S2. Spatial distribution of the first detected severe fever with thrombocytopenia syndrome cases by prefecture in Japan, 2013–2025.	7](#_Toc234962039)

[Figure S3. Geographic distribution of overall number of severe fever with thrombocytopenia syndrome cases by prefecture in Japan, 2013–2025.	8](#_Toc234962040)

[Figure S4. Nationwide weekly trends in severe fever with thrombocytopenia syndrome cases and meteorological factors in Japan, 2013–2025.	9](#_Toc234962041)

[Figure S5. Boxplot of weekly severe fever with thrombocytopenia syndrome cases and meteorological factors in Japan, 2013–2025.	10](#_Toc234962042)

[Figure S6. Sensitivity analysis of weekly mean minimum and maximum temperatures vs. mean temperature.	11](#_Toc234962043)

[Table S3. Sensitivity analysis results using alternative temperature metrics.	12](#_Toc234962044)

[Figure S7. Sensitivity analysis between severe fever with thrombocytopenia syndrome risk and meteorological, non-meteorological factors in west Japan.	13](#_Toc234962045)

[Table S4. Maximum RRs and relative risks at the 5^th^ and 95^th^ percentiles, with their corresponding exposure values for the sensitivity analysis between each factor and severe fever with thrombocytopenia syndrome risk in west Japa.	14](#_Toc234962046)

[Figure S8. Importance of the variables on SFTS incidence based on ΔAIC after removal of the variable.	15](#_Toc234962047)

[Table S5. AIC results for model construction.	16](#_Toc234962048)

[Figure S9. Geographical distribution of all the factors in Japan, 2013–2025.	17](#_Toc234962049)

# Table S1. Definition of region among Japanese prefectures

| **East/West Japan** | **Region** | **Prefecture** |
| --- | --- | --- |
| East | Hokkaido | Hokkaido |
| East | Tohoku | Aomori, Iwate, Miyagi, Akita, Yamagata, Fukushima |
| East | Hokuriku | Niigata, Toyama, Ishikawa, Fukui |
| East | Tokai | Gifu, Shizuoka, Aichi, Mie |
| West | Kinki | Shiga, Kyoto, Osaka, Hyogo, Nara, Wakayama |
| West | Chugoku | Tottori, Shimane, Okayama, Hiroshima, Yamaguchi |
| West | Shikoku | Tokushima, Kagawa, Ehime, Kochi |
| West | Kyushu | Fukuoka, Saga, Nagasaki, Kumamoto, Oita, Miyazaki, Kagoshima |
| West | Okinawa | Okinawa |

# Table S2. Variables, data sources, and their temporal resolution used in the study.

| **Category** | **Variable** | **Period** | **Temporal scale** | **Source** |
| --- | --- | --- | --- | --- |
| Outcome | SFTS cases | 2013–2025 | Weekly | National surveillance data from the National Institute of Infectious Diseases (IDWR: https://id-info.jihs.go.jp/surveillance/idwr/index.html) |
| Meteorological factors | Mean, minimum, and maximum temperatures (°C) | 2013–2025 | Weekly | ERA5-land: <https://cds.climate.copernicus.eu/datasets/reanalysis-era5-land?tab=download> |
|  | Total precipitation (mm) |  |  |  |
|  | Mean relative humidity (%) |  |  |  |
|  | Mean atmospheric pressure (hPa) | 2013–2025 | Weekly | Japan Meteorological Agency (<https://www.data.jma.go.jp/stats/etrn/>) |
|  | Mean wind speed (m/s) |  |  |  |
|  | Total sunshine duration (hours) |  |  |  |
| Land use factors | Forest coverage percentage (%) | 2012, 2017,2022 | Yearly | The Forestry Agency of Japan  (<https://www.rinya.maff.go.jp/j/keikaku/genkyou/index2.html>) |
|  | Agricultural land coverage (%) | 2013–2025 | Yearly | Ministry of Agriculture, Forestry and Fisheries  (https://www.e-stat.go.jp/) |
| Altitude | Mean altitude (m) | Static | Static | Geospatial Information Authority of Japan |
| Demographic factors | Population size | 2013–2025 | Yearly | Statistics Bureau of Japan (https://www.e-stat.go.jp/) |
|  | Proportion of population aged ≥65 years | 2013–2025 | Yearly |  |
|  | Prefecture area | Static | Static | Ministry of Agriculture, Forestry and Fisheries  (https://www.e-stat.go.jp/) |

# Figure S1. Correlation matrix of the meteorological and non-meteorological factors included in the analysis.

# Figure S2. Spatial distribution of the first detected severe fever with thrombocytopenia syndrome cases by prefecture in Japan, 2013–2025.

# Figure S3. Geographic distribution of overall number of severe fever with thrombocytopenia syndrome cases by prefecture in Japan, 2013–2025.

# Figure S4. Nationwide weekly trends in severe fever with thrombocytopenia syndrome cases and meteorological factors in Japan, 2013–2025.

# Figure S5. Boxplot of weekly severe fever with thrombocytopenia syndrome cases and meteorological factors in Japan, 2013–2025.

# Figure S6. Sensitivity analysis of weekly mean minimum and maximum temperatures vs. mean temperature.

# Table S3. Sensitivity analysis results using alternative temperature metrics.

| **Temperature metric** | **Reference Temperature(°C)** | **Maximum RR** | | **Extreme low at the 5^th^ percentile** | | **Extreme high at the 95^th^ percentile** | | **AIC** |
| --- | --- | --- | --- | --- | --- | --- | --- | --- |
|  |  | **Value** | **RR (95% CI)** | **Value** | **RR (95% CI)** | **Value** | **RR (95% CI)** |  |
| Mean temperature | 13.5 | 21.8 | 1.78 (1.26–2.52) | -0.6 | 0.10 (0.04–0.22) | 26.2 | 1.63 (0.98–2.73) | 8134.91 |
| Minimum temperature | 10.2 | 19.5 | 1.99 (1.38–2.85) | -3.7 | 0.08 (0.04–0.18) | 23.7 | 1.85 (1.10–3.10) | 8126.02 |
| Maximum temperature | 17.0 | 23.9 | 1.35 (1.00–1.83) | 2.7 | 0.16 (0.07–0.36) | 29.3 | 1.21 (0.75–1.96) | 8149.16 |

# Figure S7. Sensitivity analysis between severe fever with thrombocytopenia syndrome risk and meteorological, non-meteorological factors in west Japan.

Note: West Japan was defined as shown in Table S1 (n = 1,141 cases).

# Table S4. Maximum RRs and relative risks at the 5^th^ and 95^th^ percentiles, with their corresponding exposure values for the sensitivity analysis between each factor and severe fever with thrombocytopenia syndrome risk in west Japan.

| **Variable** | **Reference value** | **Maximum RR** | | **Extreme low at the 5^th^ percentile** | | **Extreme high at the 95^th^ percentile** | |
| --- | --- | --- | --- | --- | --- | --- | --- |
|  |  | **Value** | **RR (95% CI)** | **Value** | **RR (95% CI)** | **Value** | **RR (95% CI)** |
| Mean temperature (°C) | 15.2 | 20.7 | 1.33 (1.01–1.73) | 2.7 | 0.17 (0.09–0.30) | 26.8 | 1.03 (0.62–1.68) |
| Precipitation (mm) | 37.9 | 147.0 | 1.21 (0.97–1.50) | 2.4 | 0.88 (0.81–0.96) | 120.5 | 1.20 (1.00–1.44) |
| Atmospheric pressure (hPa) | 1011.3 | 1027.1 | 1.50 (1.08–2.10) | 1000.6 | 0.76 (0.61–0.95) | 1021.3 | 1.29 (1.05–1.60) |
| Relative humidity (%) | 76.8 | 66.4 | 1.05 (0.86–1.27) | 65.7 | 1.05 (0.85–1.29) | 88.1 | 0.83 (0.67–1.04) |
| Sunshine duration (hours) | 39.2 | 91.7 | 1.02 (0.73–1.42) | 14.7 | 0.99 (0.85–1.16) | 67.7 | 1.01 (0.84–1.21) |
| Wind speed (m/s) | 2.8 | 14.0 | 1.25 (0.45–3.49) | 1.6 | 0.98 (0.87–1.09) | 4.6 | 1.04 (0.88–1.22) |
| Forest coverage (proportion) | 0.65 | 0.84 | 6.34 (4.94–8.15) | 0.45 | 0.15 (0.12–0.19) | 0.78 | 3.58 (3.01–4.25) |
| Mean altitude (m) | 298.7 | 143.3 | 5.18 (3.96–6.77) | 131.0 | 5.04 (3.89–6.53) | 462.1 | 0.73 (0.55–0.95) |
| Dry agricultural land coverage (proportion) | 0.04 | 0.04 | 1.00 (0.90–1.11) | 0.01 | 0.96 (0.84–1.11) | 0.09 | 0.70 (0.53–0.92) |
| Population aged ≥65 years (proportion) | 0.22 | 0.25 | 1.13 (0.98–1.31) | 0.17 | 0.57 (0.47–0.69) | 0.31 | 0.78 (0.65–0.93) |

# Figure S8. Importance of the variables on SFTS incidence based on ΔAIC after removal of the variable.

# Table S5. AIC results for model construction.

| **Model** | **Components included** | **AIC** |
| --- | --- | --- |
| Seasonality model | $\log\left( \mu_{\left\{ i,t \right\}} \right)= s\left( week_{t}, bs=\text{cc}, k=6 \right)+ s\left( year_{t}, k=3 \right)+ s\left( pref_{i}, bs=\text{re} \right)+\log\left( population_{\left\{ i,t \right\}} \right)$ | 8963.31 |
| Climatic-only model | $\log\left( \mu_{\left\{ i,t \right\}} \right)= s\left( mean_{temp_{\left\{ i,t \right\}}}, k=4 \right)+ s\left( RH_{\left\{ i,t \right\}}, k=3 \right)+ s\left( precip_{\left\{ i,t \right\}}, k=3 \right)+ s\left( sunshine_{\left\{ i,t \right\}}, k=4 \right)+ s\left( pressure_{\left\{ i,t \right\}}, k=4 \right)+ s\left( wind_{\left\{ i,t \right\}}, k=7 \right)+ s\left( week_{t}, bs=\text{cc}, k=6 \right)+ s\left( year_{t}, k=3 \right)+ s\left( pref_{i}, bs=\text{re} \right)+\log\left( population_{\left\{ i,t \right\}} \right)$ | 8678.17 |
| Final full model | $\log\left( \mu_{\left\{ i,t \right\}} \right)= s\left( mean_{temp_{\left\{ i,t \right\}}}, k=4 \right)+ s\left( RH_{\left\{ i,t \right\}}, k=3 \right)+ s\left( precip_{\left\{ i,t \right\}}, k=3 \right)+ s\left( sunshine_{\left\{ i,t \right\}}, k=4 \right)+ s\left( pressure_{\left\{ i,t \right\}}, k=4 \right)+ s\left( wind_{\left\{ i,t \right\}}, k=7 \right)+ s\left( forest_{i}, k=5 \right)+ s\left( altitude_{i}, k=6 \right)+ s\left( crop_{i}, k=4 \right)+ s\left( age{65}_{i}, k=3 \right)+ s\left( week_{t}, bs=\text{cc}, k=6 \right)+ s\left( year_{t}, k=3 \right)+ s\left( pref_{i}, bs=\text{re} \right)+\log\left( population_{\left\{ i,t \right\}} \right)$ | 8134.92 |

# Figure S9. Geographical distribution of all the factors in Japan, 2013–2025.
